# Advancing Discussions Using a Video-Based Support Tool About End-of-Life Care (ADVISE): Protocol for a Single-Center Randomized Controlled Trial

**DOI:** 10.2196/87690

**Published:** 2026-06-18

**Authors:** Mokunfayo O Fajemisin, Ashna S Karpe, Stephanie Martinez Ugarte, Martin L Blakely, Gina Khraish, Shreyans Sanghvi, Jessica L Lee, Lillian S Kao, Thaddeus J Puzio

**Affiliations:** 1 The University of Texas Health Science Center at Houston Houston, TX United States

**Keywords:** decision aid, older people, trauma, video, cardiopulmonary resuscitation, code status

## Abstract

**Background:**

More than 950,000 adults over 65 years of age are hospitalized each year after injury. Recommended care for this vulnerable group includes discussion of their goals for life-sustaining care (“code status”) upon admission, but most older patients do not participate in adequate code status discussions. Randomized trials have demonstrated that video-based interventions are associated with better patient knowledge and improved alignment of patient preferences for life-sustaining treatment; however, there are limited data on the effectiveness of these interventions in acutely injured patients.

**Objective:**

This trial will evaluate the effectiveness and implementation considerations of a video conversation aid to enhance communication among trauma care providers, patients, and surrogate decision-makers regarding cardiopulmonary resuscitation (CPR) and code status decisions.

**Methods:**

This is a single-center randomized controlled trial evaluating the use of a video conversation aid to facilitate discussions of code status. The video intervention will be compared to usual care in the largest feasible pilot randomized controlled trial with the goal of estimating its benefit for patient-centered outcomes. All trauma patients aged 65 years and older admitted following injury are eligible. Incarcerated people, patients with existing do-not-resuscitate or do-not-intubate orders, patients admitted while in hospice care, and patients not expected to survive over 24 to 48 hours will be excluded. The primary outcome is transitions in code status. Secondary outcomes will include perceived success of CPR, video acceptability and effectiveness, knowledge of life-sustaining care outcomes and complications following CPR, participant desire for more information regarding life-sustaining care, hospital-free days, intensive care unit–free days, ventilator-free days, discharge disposition, decisional conflict score, and rate of interventional procedures (eg, tracheostomy, feeding tube placement, and dialysis initiation).

**Results:**

The ADVISE (Advancing Discussions Using a Video-Based Support Tool About End-of-Life Care) trial was funded in February 2025 via the Learning Healthcare System grant from the University of Texas Health Science Center at Houston. Enrollment began on March 17, 2025, and it is estimated to conclude on May 1, 2026.

**Conclusions:**

The ADVISE trial builds on previous research on older trauma patients and advanced care planning using a video intervention to enhance code status discussions. We anticipate that our video aid tool will enhance patients’ and surrogate decision-makers’ knowledge of advanced care discussions.

**Trial Registration:**

ClinicalTrials.gov NCT06804226; https://clinicaltrials.gov/study/NCT06804226

**International Registered Report Identifier (IRRID):**

DERR1-10.2196/87690

## Introduction

Traumatic injury is responsible for more than 950,000 hospitalizations in adults over 65 years of age each year [1]. The American College of Surgeons Best Practices Guidelines advise that care for this vulnerable group should include a discussion regarding goals for life-sustaining care (sometimes referred to as “code status”) upon admission [2]. However, there are multiple barriers to having effective code status discussions with older injured patients, and most injured older adults do not receive this counseling [3]. Randomized trials have demonstrated that video-based interventions are associated with deeper patient understanding and patient choices that avoid acute cardiopulmonary resuscitation (CPR) [4]. There are limited data on the effectiveness of video-based interventions in the acutely injured population.

One identified barrier is the lack of understanding on the part of many patients of the associated risks and benefits of CPR and preconceived unrealistic expectations of its success [5,6]. As one of the largest physician barriers to engaging in code status discussions is time constraints, this lack of knowledge regarding CPR is often never addressed [7-9]. When discussions do occur, physicians often use medical jargon and infrequently provide prognostic information [7]. As a result, code status discussions are often brief and ineffective at communicating and increasing understanding of life-sustaining interventions, limiting the ability of patients and surrogate decision-makers to make informed decisions about critical care goals [8-10].

This multidisciplinary, collaborative research project will evaluate the effectiveness of a video aid to improve communication and patient-centered outcomes in older injured adults. This video conversation aid is not intended to replace code status discussions between patients and health care providers but to promote these discussions. The goal is to overcome identified barriers to adequate code status discussions by providing a clear message with no medical jargon; supplying accurate prognostic information; and, ultimately, enhancing informed consent so that patients can ensure that their care choices align with their preferences. We hypothesize that our video aid will increase the rate of code status transitions, with more patients documenting their code status. Additionally, we hypothesize that our video aid will improve knowledge of life-sustaining care and increase code status discussions.

## Methods

The trial was designed in accordance with the SPIRIT (Standard Protocol Items: Recommendations for Interventional Trials) 2013 guidelines [11].

### Study Setting

Our trial will be conducted at the Red Duke Trauma Institute at Memorial Hermann–Texas Medical Center. This center is 1 of only 2 adult level 1 trauma centers in the city of Houston, Texas, and serves over 7 million people. Approximately 200 trauma patients aged ≥65 years are admitted per month, leading to over 2800 total older adult inpatients per year.

### Study Population

All English- or Spanish-speaking patients aged 65 years or older admitted to any level of care following traumatic injury will be screened for study eligibility. As this is a pilot trial, we will have minimal exclusion criteria except for (1) incarcerated people, (2) existing do-not-resuscitate (DNR) or do-not-intubate (DNI) orders, (3) patients admitted while in hospice care, and (4) patients not expected to survive over 24 to 48 hours. Incarcerated individuals are excluded due to being a vulnerable population in a study that waives informed consent. Patients with established DNR or DNI orders have already made decisions regarding their code status; therefore, including them would impede our ability to assess the intervention’s effect on code status decision-making. Auditory and visual impairments are not barriers to participation. The code status video includes subtitles, and patients have the option to have the research assistant administer the study survey verbally. All patients who meet the eligibility criteria will be offered enrollment within 48 hours whenever feasible.

### Intervention Group

A video conversation aid that provides information about life-sustaining care and encourages further discussions on goals of care with a health care provider will be shown to patients or their surrogate decision-makers upon admission or within 48 hours, whichever is more feasible. The video aid is available in both English and Spanish. Surrogate decision-makers are required for patients who lack decision-making capacity, specifically, those unable to understand their medical condition, appreciate the consequences of treatment options, or engage in rational decision-making [2]. This video will be administered by a research assistant who is not part of the patient’s care team.

The video script was created by a multidisciplinary team encompassing emergency medicine, palliative care, geriatric medicine, internal medicine, and trauma surgery along with content experts in health literacy and medical ethics. An iterative approach was used, and script development followed the International Patient Decision Aids Standards Collaboration quality criteria as a framework for the creation of the video decision aid. The goal of the video was to provide standardized information for code status discussions. After providers reached consensus, the script was revised based on feedback from community patient representatives and patients from a standardized patient advocacy group at MD Anderson Cancer Center. Adjustments were made to simplify the medical language and better accommodate patients with low health literacy. The final video was produced by the Kashu animation company at a fourth-grade reading level in both English and Spanish. An animated format was chosen based on feedback from community groups indicating that it was the least distressing to patients. After the animated video was created, it was reassessed by the community group for final feedback and found to be both easy to follow and not distressing to patients. After administration of the code status video, if patients choose to change their code status or have additional questions, they may discuss them with their primary inpatient medical care team. For patients who experience distress, additional support is available through the supportive medicine team and chaplaincy services.

### Usual Care Group

Patients and/or surrogate decision-makers will participate in discussions surrounding life-sustaining care at health care providers’ discretion. This task is most often completed by resident physicians, but there is no standardization regarding participants or timing.

### Procedures and Outcomes

A research assistant will administer a survey to each participant at discharge either collected verbally or completed electronically via QuestionPro. Patients’ decision-making capacity is determined by the primary clinical team. For patients who die during admission, surrogate decision-makers will be contacted after discharge. Demographics, injury characteristics, length of stay, preexisting advanced directives, and disposition will be collected using the institutional trauma registry. Each participant will be assigned a study-specific number.

The primary outcome will be the rate of transition in code status. Prior institutional data have shown that a minority of patients have advanced directives or code status discussions with their providers; therefore, this serves as a proxy for increased patient or surrogate decision-maker knowledge.

Secondary outcomes will include perceived success of CPR, knowledge of life-sustaining care outcomes and complications following CPR, and participant desire for more information regarding life-sustaining care. These will all be assessed using a Likert scale via a survey at the time of discharge. Decisional conflict score will also be assessed at the time of discharge using an electronic survey. Hospital-free days, intensive care unit (ICU)–free days, ventilator-free days, discharge disposition, and rate of interventional procedures (eg, tracheostomy, feeding tube placement, and dialysis initiation) will be collected from the electronic medical record.

### Ethical Considerations

A waiver of written consent was granted by the University of Texas Health Science Center at Houston (UTHealth Houston) Institutional Review Board based on a category III exemption and Title 45 of the Code of Federal Regulations part 46 as the intervention does not pose more than minimal risk to patients or caregivers [12]. The informative video can be considered a “benign behavioral intervention that is brief in duration, harmless, painless, not physically invasive, not likely to have a significant adverse lasting impact on the subjects, and there is no reason to think the subjects will find the interventions offensive or embarrassing” [13]. Additionally, there is no standard of care regarding optimal patient decisional counseling, and this intervention is a reasonable strategy for usual care. Therefore, we will obtain verbal consent for enrollment, and patients will receive a letter of intent prior to survey completion.

The institutional review board of McGovern Medical School at UTHealth Houston approved the study protocol on January 21, 2025. Enrollment began on March 17, 2025, and is scheduled to continue for 18 months. Information and data from patients included in the study will be accessed only by designated study personnel, and the data will be collected in a secure database and deidentified. The ADVISE (Advancing Discussions Using a Video-Based Support Tool About End-of-Life Care) project (NCT06804226) was registered on January 31, 2025, on ClinicalTrials.gov.

### Randomization and Blinding

This is a single-center pilot randomized trial. Randomization will be performed in a 1:1 ratio between the intervention and usual care groups using REDCap (Research Electronic Data Capture; Vanderbilt University). We plan to stratify based on whether the patient or a surrogate decision-maker watches the video aid. Allocation will be performed using the REDCap randomization module, after which a research assistant will administer the video intervention. Thus, providers will be blinded to treatment group. The project statistician will be blinded to group assignment.

### Sample Size Calculation

We plan to conduct the largest feasible randomized controlled trial (RCT) with enrollment lasting 18 months. We conservatively estimate that at least 15 patients will be enrolled per month, with the potential for 270 randomized patients during the study period. A Bayesian analysis will be performed to estimate the probability of treatment benefit. This estimate is based on recommendations for pilot trials with limited sample size and power in frequentist analyses to identify conclusive treatment effects [14].

### Data Analysis Plan

Analysis will be performed on a per-patient basis. For patients who are incapacitated and unable to consent for themselves, their surrogate decision-makers will serve as the participant for analysis. The number of screened patients and reasons for exclusion will be reported. Protocol violations and reasons for those violations will be reported and detailed. All analyses will be based on the intention-to-treat principle. Regarding the primary end point, we will estimate the risk ratio between the intervention and usual care groups together with a 95% Wald CI. For other outcome variables, we will implement 2-group comparisons using the Wald test for dichotomous variables and the Wilcoxon rank-sum test for continuous variables. ICU-free days and ventilator-free days refer to the number of days during admission without intensive care or a ventilator [15]. In case of a death event, these variables are set to a value of 0. The principal investigator will audit data collection to ensure that the data are accurate, consistent, complete, and reliable.

For the primary end point, in addition to the frequentist inference, we will conduct a Bayesian analysis to enhance the knowledge gained from the study. Bayesian analysis enhances the clinical decision-making process by integrating existing evidence with data from a new study to estimate the probability of an outcome of interest. In contrast, frequentist analysis assesses the likelihood of observing the obtained results or more extreme outcomes. For each group, we will use the conjugate β prior for the probability of transition in code status. The parameter values for β distribution will be chosen to center a symmetrical distribution at 0.5, and the corresponding distribution for the risk ratio will be centered at 1 with a credible interval of 0.5 to 2.0, resulting in a neutral prior [16]. Additionally, we will perform sensitivity analysis by identifying suitable β prior distributions to generate enthusiastic and skeptical priors. We will use the JAGS software (version 4.3.2) for Bayesian analysis. All other statistical analyses will be performed using the SAS software (version 9.4; SAS Institute).

We plan to perform several subgroup analyses, including comparisons based on race, gender, health literacy assessed via the validated Brief Health Literacy Screening Tool, and surrogate decision-maker vs patient watching the video [17]. The Brief Health Literacy Screening Tool has been incorporated into the study and will be administered simultaneously.

## Results

The ADVISE trial was funded in February 2025 via the Learning Healthcare grant from UTHealth Houston. The ADVISE RCT began enrollment on March 17, 2025. It is estimated to conclude on May 1, 2026. As of April 2026, the trial has enrolled 270 patients. We plan to submit the results in abstract form by July 2026 and publish in early 2027.

## Discussion

### Expected Findings

We hypothesize that our video aid will increase the rate of code status transitions, with more patients documenting their code status. Additionally, we hypothesize that our video aid will improve knowledge of life-sustaining care and increase code status discussions. Video-based interventions to inform code status have been reported to be effective in empowering patients to make informed choices regarding advanced care and improving knowledge; studies of these interventions are often limited to oncology, and outpatient settings [18-23]. Previous single-center studies conducted in medical ICUs have found video interventions beneficial in improving resident comfort with conducting code status discussions [23]. Additionally, in another study, patients and surrogate decision-makers who received a video intervention exhibited a better understanding of CPR, resuscitation terminology, and medical resuscitation options [22]. A single-center RCT conducted on patients admitted for a medical reason found that those receiving a video intervention were more likely to choose DNR and DNI status over full code status [24]. Video conversation aids to improve code status discussions have not been evaluated in older trauma patients.

Proposed benefits of a video-based intervention include overcoming time constraints and discomfort with difficult conversations on the part of the health care team; video aids also allow for standardization of the information conveyed and adjustment for low health literacy. On the other hand, video-based interventions may not overcome the problem of limited prognostic information. It is unknown whether a video-based intervention would prompt earlier and more comprehensive discussions about life-sustaining care or inhibit them [22].

The proposed project is innovative in that it aims to evaluate and implement an intervention in a unique patient population—older trauma patients—that has not been previously studied. This trial will evaluate the effectiveness of our video intervention by measuring the rate of code status transitions and assessing baseline knowledge of CPR and code status. We expect that a minority of patients have advanced directives. Additionally, most patients have low baseline knowledge of life-sustaining care.

### Limitations

One major limitation of our study is that our video intervention will only be offered in English or Spanish, and thus, our results are not applicable to patients who speak other languages. Limited English-language proficiency has been associated with reduced decision-making ability and poor health care provider–patient communication [25]. The Decisional Conflict Scale used in this study is administered at the time of discharge instead of immediately following the decision, which potentially introduces recall bias. A validated tool for CPR knowledge is not used in this study; instead, CPR knowledge was contingent on knowledge of CPR survival statistics. Another limitation is that patients who proactively choose full code status are not being tracked, and this is not reflected in the primary outcome. Finally, the results may not be generalizable as this is a single-center trial.

### Conclusions

In summary, the ADVISE trial builds on previous research on older trauma patients and advanced care planning using a video intervention to enhance code status discussions in this specific patient population. We anticipate that our video conversation aid will be an effective means to increase patient and surrogate decision-maker knowledge regarding goals for life-sustaining care and will empower patients to have a discussion with their providers regarding their health care goals.

## Abbreviations

ADVISE: Advancing Discussions Using a Video-Based Support Tool About End-of-Life Care
CPR: cardiopulmonary resuscitation
DNI: do not intubate
DNR: do not resuscitate
ICU: intensive care unit
RCT: randomized controlled trial
REDCap: Research Electronic Data Capture
SPIRIT: Standard Protocol Items: Recommendations for Interventional Trials
UTHealth Houston: University of Texas Health Science Center at Houston

## Data Availability

Deidentified data will be available on reasonable request from the corresponding author.
